# Antibacterial, Antioxidant, and *in silico* NADPH Oxidase Inhibition Studies of Essential Oils of *Lavandula dentata* against Foodborne Pathogens

**DOI:** 10.1155/2023/9766002

**Published:** 2023-02-11

**Authors:** Youness El Abdali, Ghada Beniaich, Adil M. Mahraz, Abdelfattah El Moussaoui, Yousef A. Bin Jardan, Mohamed Akhazzane, Mohamed Chebaibi, Hiba-Allah Nafidi, Noureddine Eloutassi, Mohammed Bourhia, Abdelhak Bouia

**Affiliations:** ^1^Laboratory of Biotechnology, Environment, Agri-Food and Health, Faculty of Sciences Dhar El Mahraz, Sidi Mohamed Ben Abdellah University, P.O. Box 1796, Atlas, Fez 30000, Morocco; ^2^Engineering Laboratory of Organometallic, Molecular Materials and Environment, Faculty of Sciences Dhar El Mahraz, Sidi Mohamed Ben Abdellah University, P.O. Box 1796, Atlas, Fez 30000, Morocco; ^3^Department of Pharmaceutics, College of Pharmacy, King Saud University, Riyadh, Saudi Arabia; ^4^Biomedical and Translational Research Laboratory, Faculty of Medicine and Pharmacy, University of Sidi Mohamed Ben Abdellah, BP 1893, Km 22, Road of Sidi Harazem, Fez, Morocco; ^5^Department of Food Science, Faculty of Agricultural and Food Sciences, Laval University, Quebec City 2325, QC G1V0A6, Canada; ^6^Laboratory of Pedagogy and Technological Innovation, Regional Centre of Education and Formation Professions, Fez 30000, Morocco; ^7^Laboratory of Chemistry and Biochemistry, Faculty of Medicine and Pharmacy, Ibn Zohr University, Laayoune 70000, Morocco

## Abstract

Food is always subjected to microbial infection and lipid peroxidation, which frequently leads to serious food intoxications. In the present study, essential oils (EOs) extracted from *Lavandula dentata* Moroccan species and its major component (linalool) were chemically characterized and their antioxidant potential and antibacterial properties against foodborne pathogenic bacteria were examined. EOs phytochemical profile was carried out using gas chromatography-mass spectrometry analysis (GC-MS). The antioxidant potential was evaluated, *in vitro*, by use of the *β*-carotene discoloration assay and *in silico* vs. NADPH oxidase enzymatic complex as an antioxidant marker. The antibacterial proprieties were assessed by use of minimal inhibitory concentration (MIC) and disc diffusion methods, against Gram (−) bacteria (*Pseudomonas aeruginosa*, *Salmonella enterica*, and *Escherichia coli*) and Gram (+) bacteria (*Bacillus subtilis* and *Staphylococcus aureus*). Linalool (49.71%) was the major component among the eighteen components identified in *Lavandula dentate* EO, followed by camphor (14.36%) and borneol (8.21%). The studied EO and linalool compounds showed important antioxidant activity through the *β*-carotene discoloration test with IC_50_ values of 35.72 ± 1.21 mg/mL and 30.32 ± 1.23 mg/mL, respectively. Among all the analyzed compounds of lavender EOs, thymol, carvacrol, and *α*-terpineol were the most active compounds against NADPH oxidase with a glide score of −6.483, −6.17, and −4.728 kcal/mol, respectively. 2D and 3D views showed the formation of hydrogen bonds between the most active compounds and the active site of NADPH oxidase. The antibacterial data showed a significant activity of *Lavandula dentata* essences against tested foodborne pathogenic bacteria, especially against *S. aureus* and *B. subtilis*. Linalool proved active toward the same bacteria and had closer activity to that of lavender essential oil. In light of the obtained findings, the essential oil of *Lavandula dentata* Moroccan species can be used in the packaging sector as a promising natural food conservative to limit lipid oxidation and treat foodborne infections.

## 1. Introduction

Antibiotic resistance is the leading cause of food poisoning outbreaks. Infections caused by resistant microbes can occur after eating contaminated food. *Campylobacter*, *Salmonella*, methicillin-resistant*Staphylococcus aureus* (MRSA), and some *Escherichia coli* strains are among antibiotic-resistant zoonotic bacteria that may infect humans through food and lead to treatment failure. Furthermore, the commensal bacterial flora may serve as a source of resistance genes that can be transmitted to resistant species and involve in illness in people and animals [1].

In fact, the emergence of antibiotic-resistant bacteria, both zoonotic and food-borne, is mainly due to the overuse of animal antibiotics in livestock and animal husbandry, as treatment or growth promoters. Animal foodstuffs contaminated with these microorganisms are potential vectors for human infections [2, 3]. One of the greatest threats to human health today is antibiotic resistance, which presents a significant obstacle to alternative drug research initiatives throughout the world [4]. Since bacteria cannotresist EOs, they represent potential alternatives to control antibiotic resistance in this regard [5].

Free radicals can harm the body's cells, causing damage to various oxygen substrates, DNA, proteins, fatty acids, and lipids [6, 7]. Today, the presence of antioxidants in food has become essential for quality and food safety. As synthetic antioxidants have negative effects on human health, aromatic plants can be used as a source of antioxidants and natural antibacterial agents for the agri-food industry, especially in food packaging systems [8, 9]. During the last few years, natural products have aroused great interest as medications, pharmaceutical products, cosmetics, and food additives [10].

Widely used in prophetic medicine, as well as in traditional medicine in Morocco, *Lavandula dentata* (lavender) possesses a large spectrum of biological activities, including sedative, antidepressant, anti-inflammatory, antioxidant, antibacterial, and antifungal properties [11]. However, there is a lack of research and information on the activities of Moroccan varieties, especially against food-borne pathogens resistant to antibiotics, which deserve to be examined in order to expand the spectrum of biological activities and applications of Moroccan *Lavandula dentata*. It is thus fitting that we were interested in investigating this emblematic plant of the Moroccan pharmacopoeia, through phytochemical characterization of its essential oils and their antioxidant (*in vitro* and *in silico*) and antibacterial activities against antibiotic-resistant pathogens.

## 2. Materials and Methods

### 2.1. Chemicals

Butylated hydroxytoluene (BHT), dimethylsulfoxide (DMSO), resazurin, and *β*-carotene were obtained from Sigma-Aldrich (Germany). Antibiotics and bacterial culture media were obtained from Biokar Diagnostics (France).

### 2.2. Plant Materials

**Figure 1:**
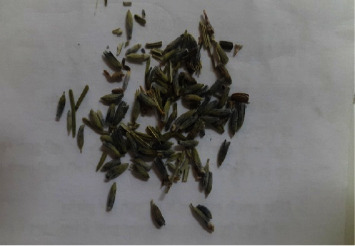
Flowering tops of *Lavandula dentate* L.

The flowering tops of *Lavandula dentata* were used in the present study for testing (Figure 1). The plant specimens were collected from around Imouzzer Kandar city (33°44′ N, 5°01′ W at 1300 m altitude) of Morocco at the end of May, 2020. The samples were vouchered under the number DL78/24811, after being identified by a botanist. The collected samples were cleaned and dried for 15 days in a shaded and ventilated area before being subjected to extraction.

### 2.3. Extraction of Essential Oil

A total of 100 g of dried aerial parts were hydrodistillated with 1 L of distilled water using a clevenger-type apparatus for 3 h as documented elsewhere [12]. By use of anhydrous sodium sulfate, EOs were stored in darkness at 4 F02D 5°C in the dark until use. The yield of EOs was calculated and given in percentage (v/w).

### 2.4. Chemical Characterization of Essential Oil

The chemical composition of *L. dentata* EO was investigated using gas chromatography TQ8040 NX (Shimadzu, Tokyo, Japan) coupled with a triple quadrupole, tandem mass spectrometer (GC-MSMS). The EO chemical compounds were identified using an apolar, capillary RTxi-5 Sil MS column (30.00 m long, 00.25 mm inside diameter, and a film thickness of 0.25). 200°C and 280°C were the source and interface temperatures, respectively. Helium was employed as the carrier gas (with an injection volume of 1 *μ*L). The oven temperature was programmed to start at 50°C for 2 minutes and it increased following two rises, rise 1 was set to 5°C/min to 160°C for 2 min and rise 2 was set to 5°C/min to 280°C for 2 min. The injection was in mode split (split opening at 4 min, with an injection temperature of 250°C and a pressure of 37.10 kPa). The composition of EO was presented as a proportion of the overall peak area. EO phytochemicals in EOs were identified by comparing the resulted retention indices referring to those of the literature database [13].

### 2.5. *In Vitro* Antioxidant Activity of Essential Oil

The antioxidant propriety of the extracted EO and its major compound (linalool) was evaluated using the *β*-carotene/linoleic acid assay [14]. In this assay, 1 mL of a chloroformic solution of *β*-carotene (1 mg/10 mL) was mixed with 20 mg of linoleic acid and 200 mg of Tween 40. Using a rotary evaporator, the chloroform was evaporated at 40°C for 5 minutes. Afterward, 50 mL of hydrogen peroxide was slowly added to the residue obtained with continuous stirring until an emulsion was formed. A volume of 5 mL of the obtained emulsion was added to all tubes containing 0.20 mL of ethanolic solution of EO and linalool (at various concentrations), and then, the absorbance at 470 nm was immediately measured relative to a blank not containing *β*-carotene. After incubation at 50°C for 5 minutes in a water bath, the absorbance of the tubes was measured again. BHT and ethanol were utilized as standard and negative control, respectively.

The antioxidant activity of the EO and linalool was evaluated as the percentage (%) of *β*-carotene discoloration inhibition using the following formula:

(1) $$\begin{array}{c} \beta -carotene\;antiradical\;activity\left(\%\right)=\left[\frac{\left(A_{t}-C_{t}\right)}{C_{0}-C_{t}}\right]\times 100\text{,} \end{array}$$

where *A*_*t*_ and *C*_*t*_ are the absorbance of the lavender EO and the control after 5 min of incubation, respectively. *C*_0_ is the control absorbance measured at zero minute.

The kinetics of this activity were allowed to determine the sample's concentration corresponding to a 50% inhibition of *β*-carotene discoloration (IC_50_).

### 2.6. *In Silico* Molecular Docking of Antioxidant Activity of Essential Oil

In this test, the antioxidant activity was conducted to examine the, *in silico*, inhibitory potency of *Lavandula dentata* EO against nicotinamide-adenine dinucleotide phosphate (NADPH) oxidase as an important antioxidant marker. All compounds identified in the studied EO were downloaded from the PubChem database in SDF format. Afterward, the Maestro 11.5 of the Schrödinger Software was used to prepare chemicals using the OPLS3 force field and the LigPrep tool. At a pH of 7.0 ± 2.0, ionization states generated 32 stereoisomers per ligand. The NADPH oxidase crystal structure in the Protein Data Bank was accessible through the following PDB ID : 2CDU. The structure was prepared and refined using the Protein Preparation Wizard of Schrödinger-Maestro v11.5. The minimization of the structure was conducted using the OPLS3 force field. The receptor grid was set at the following coordinates: *X* = 0.395, *Y* = 10.379, and *Z* = 53.876, where the volumetric spacing performed was 20 × 20 × 20. SP flexible ligand docking was carried out in glide of Schrödinger-Maestro v 11.5 [15].

### 2.7. Antibacterial Activity of Essential Oil

#### 2.7.1. Growth Medium and Chemicals

Tryptic soy agar (TSA) was the growth medium used for the bacterial strains in this study. It was prepared and autoclaved at 120°C for 20 min. Dimethylsulfoxide (DMSO) was used as a negative control. Disc antibiograms of vancomycin, streptomycin, and chloramphenicol were used as standard drugs.

#### 2.7.2. Bacterial Strains

The antibacterial activity of *Lavandula dentata* EO and its major compound (linalool) was tested against five bacterial strains: Gram (−) bacteria including *Pseudomonas aeruginosa* (CIP 82118), *Salmonella entérica* (CIP 8039), and *Escherichia coli* (CIP 53126) and Gram (+) bacteria including *Bacillus subtilis* (CIP 5262) and *Staphylococcus aureus* (CIP 483), which were obtained from the collection of the Pasteur Institute (Rabat, Morocco). The selected bacteria are pathogenic and frequently involved in the contamination and spoilage of foodstuffs, which may constitute a major public health problem. The purity of the studied strains was verified by macroscopic characterization (colony characteristics) and microscopic observation of smears stained with the Gram stain.

The microbial suspension was made by collecting 2 to 3 colonies from a fresh culture (aged 24 hours) and suspending them in a 0.9% NaCl solution. The optical density of the suspensions was then determined using a UV-visible spectrophotometer at a wavelength of *λ* = 625 nm (Selecta, E. U). The final bacterial suspension was approximately of 5 × 10^5^ CFU/mL [16].

#### 2.7.3. Agar Disk Diffusion Assay

First, the bacterial sensitivity was assessed using the agar disk diffusion technique, which is extensively used to assess antimicrobial activity [17]. Initially, the Petri dishes (9 cm) containing TSA medium were inoculated with 0.1 mL of fresh bacterial culture and left to solidify. Next, sterile discs (6 mm) soaked with 15 *μ*L of the test material (EO and linalool) and standard drugs were deposited on the agar surface. Finally, the zones around the disks were measured in mm after incubation at 37°C for 24 h. The test was repeated three times [18].

#### 2.7.4. Determination of the Minimal Inhibitory Concentration (MIC)

Microdilution in a 96-well microplate assay was used to investigate the MICs of the test material [17]. First, 100 *μ*L of the TSA broth was deposited in every well of the microplate. Afterward, 100 *μ*L of the EOs solution (25 mg/mL, w/v in DMSO 90%), linalool (25 mg/mL), and chloramphenicol (50 mg/mL) were deposited in the first wells. Then, excluding the last well (positive growth control), a microdilution was conducted by transferring 100 *μ*L from the first well to the second and so on (diluting the material by a factor of 1/2 in each well). Next, inoculation was performed by depositing 10 *μ*L of the microbial suspension, prepared previously, into each well except the negative control. Finally, the microplate was incubated for 24 h at 37°C. After incubation, 10 *μ*L of resazurin (6.75 mg/mL in sterile distilled water) was added to each well to read the results [19]. The microplate was placed for the second time for 2 h in an incubator at 37°C. Indeed, wells containing bacterial growth changed their color from purple to pink, while wells without bacterial growth remained purple. Consequently, the MIC value with the lowest concentration did not produce a color change [20].

### 2.8. Statistical Analysis

GraphPad Prism 8, a Microsoft Software (California, USA), was employed to calculate mean values and standard deviations. Data of all tests were compared statistically by one-way ANOVA and Tukey's test, using the same software. At *p* < 0.05, the difference was considered significant.

## 3. Results

### 3.1. Chemical Composition and Yield of *Lavandula dentata* Essential Oil

The EO obtained from *Lavandula dentata* was pale yellow-green in color with a fresh and characteristic odor and yielded 3.47% of EO. The GC/MS profile and the phytochemicals identified in EO are summarized in Table 1. The findings of the chemical analysis revealed eighteen compounds in *Lavandula dentata* EO representing 99.97% of the total oil mass. Linalool (49.71%) was the major component followed by camphor (14.36%) and borneol (8.21%). Monoterpene chemical classes constituted the major chemical groups in *L. dentata* EO (92.51%). The *β*-farnesene (0.67%) was the only sesquiterpene hydrocarbon, while other compounds were listed with a rate not exceeding 6.79% of the studied EO.

### 3.2. *In Vitro* Antioxidant Activity of *Lavandula dentata* Essential Oil

**Figure 2:**
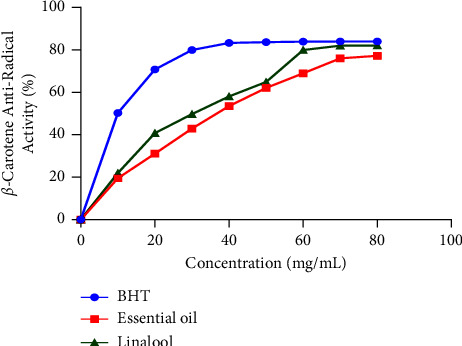
*β*-carotene antiradical activity of linalool and EO extracted from *L. dentata.*

**Figure 3:**
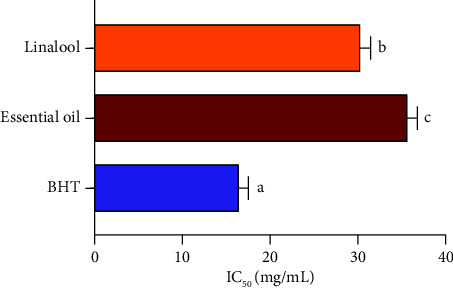
IC_50_ values (means ± SD) of antiradical activity of linalool and EO extracted from *L. dentata.* bars with different letters are significantly different (*p* < 0.05).

The capacity of lavender EO in limitation of lipid oxidation was assessed in this study by evaluating the inhibitory effect of linoleic acid oxidation in the presence of *β*-carotene as a marker. *β*-carotene usually discolors rapidly in absence of antioxidants due to free radicals released from linoleic acid [21]. The results (Figure 2) showed that *Lavandula dentata* EO exhibited an important antioxidant effect in a concentration-dependent manner, limiting a maximum of 77.2% oxidation of linoleic acid when compared to the antioxidant effect obtained with BHT (84%). Linalool showed a potent antioxidant capacity of 82.1%. The concentrations of samples required to inhibit 50% of *β*-carotene discoloration (IC_50_) are presented in Figure 3. IC_50_ values of both lavender EO and linalool were 35.72 *±* 1.21 mg/mL and 30.32 *±* 1.23 mg/mL, respectively. Notably, the IC_50_ value for the BHT standard was 16.46 *±* 1.13 mg/mL.

### 3.3. *In Silico* Molecular Docking of Antioxidant Activity of *Lavandula dentata* Essential Oil

The antioxidant effect of lavender EO was also tested, *in silico*, against NADPH oxidase enzyme complex as an antioxidant marker. As shown in Table 2, the EO compounds exhibited an inhibitory effect against NADPH oxidase expressed in free binding energy. Thymol, carvacrol, *α*-terpineol, and camphor were the most active compounds against the active site of NADPH oxidase with a glide score of −6.483, −6.17, −4.728, and −4.541 kcal/mol, respectively.

**Figure 4:**
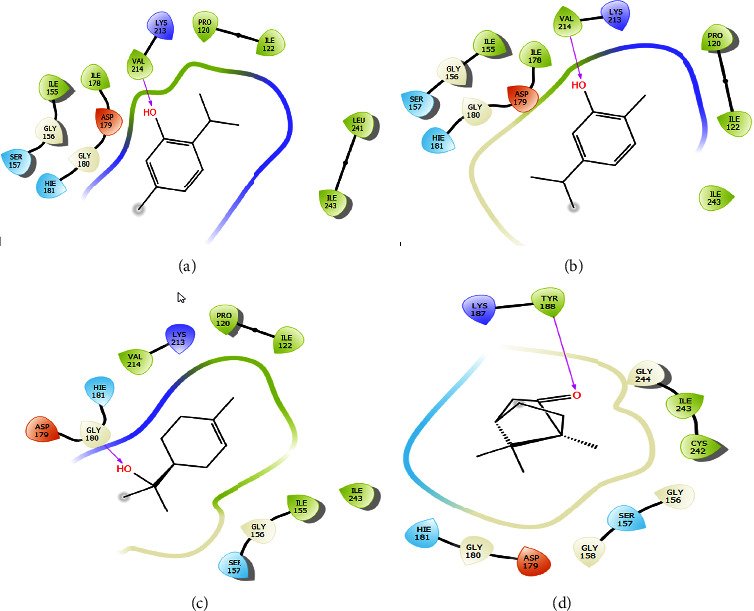
2D diagrams of ligand interactions with the active site of NADPH. (a) Thymol; (b) carvacrol; (c) *α*-terpineol; (d) camphor.

**Figure 5:**
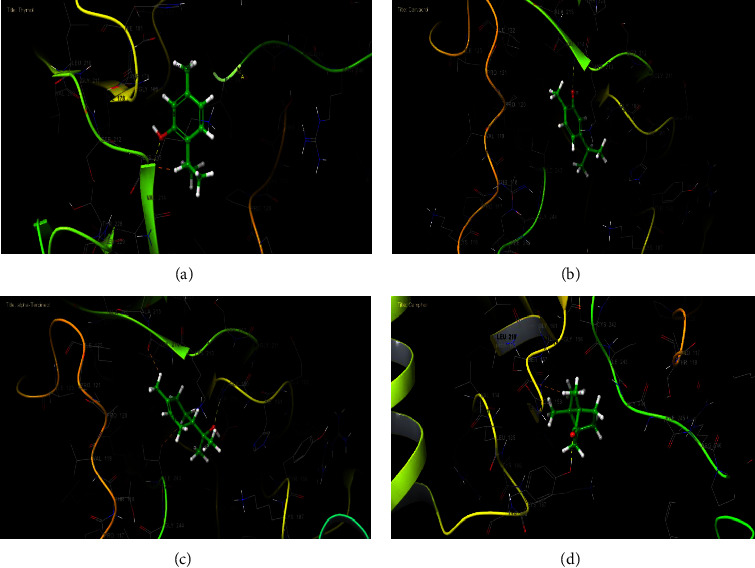
3D diagrams of ligand interactions with the active site of NADPH. (a) Thymol; (b) carvacrol; (c) *α*-terpineol; (d) camphor.

2D and 3D views of *Lavandula dentata* EO, docked in the active site of NADPH oxidase, showed the formation of hydrogen bonds between the VAL 214 residue and the OH group of thymol and carvacrol; between the OH group of *α*-terpineol and GLY 180 and between the carbonyl group of camphor and TYR 188 residue (Figures 4 and 5). These results agreed with antioxidant potency tested by the use of *β*-carotene discoloration assay (Figures 2 and 3).

### 3.4. Antibacterial Activity of *Lavandula dentata* Essential Oil

**Figure 6:**
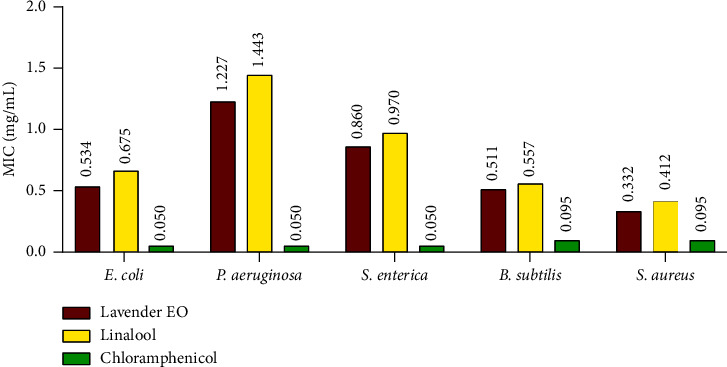
Minimal inhibitory concentration values (mg/mL) of linalool and EO extracted from *L. dentata* against bacterial strains.

The antibacterial activity of *Lavandula dentata* EO and linalool compound was studied, *in vitro,* using agar disc diffusion and minimal inhibitory concentration (MIC) methods against five food-bornepathogenic-bacterial strains (*S. enterica, S. aureus*, *P. aeruginosa, B. subtilis,* and *E. coli*), involved in food illness for both humans and animals [1]. Data obtained are summarized in Table 3 and Figure 6.

In fact, the obtained results of the growth inhibition zone scored in Tryptic soy agar demonstrated a wide antibacterial spectrum of studied EO and its major compound linalool (49.71%) against all tested strains, with different inhibition zone diameters. Both Gram (+) and Gram (−) bacteria were sensitive to lavender EO, which was particularly effective against *Escherichia coli* with a 17.08 ± 0.79 mm growth inhibition diameter, except for *Pseudomonas aeruginosa* (9.06 ± 0.63 mm), which was the least sensitive strain to the same EO. Compared to the EO, the linalool compound was more active against all tested bacterial strains marking larger diameters of growth inhibition. When subjected to treatment with the linalool compound, the Gram (+) *Bacillus subtilis* was the most sensitive of the strains tested followed by *Staphylococcus aureus* marked by a clear growth inhibition diameter of 29.00 ± 1.15 mm and 25.00 ± 1.73 mm, respectively. However, linalool showed a low activity against the Gram (−) *P. aeruginosa* (15.08 ± 0.50 mm). These results were sometimes better than those of some standard antibiotics tested such as streptomycin and vancomycin (Table 3).

The results of the bacteriostatic effectiveness of the tested samples estimated by minimum inhibitory concentration (MIC) are shown in Figure 6. According to the findings, the lowest inhibitory concentrations of lavender EO were observed for Gram (+) bacterial strains with concentrations of 0.332 mg/mL and 0.511 mg/mL in the case of *S. aureus* and *B. subtilis,* respectively. On the other hand, linalool exhibited a bacteriostatic effect close to that of lavender EO, with MIC values that ranged from 0.412 mg/mL for Gram (+) *S. aureus* to 1.443 mg/mL for Gram (−) *P. aeruginosa*, which was the least sensitive. Notably, the chloramphenicol antibiotic exhibited lower MICs against the tested strains.

## 4. Discussion

Hydrodistillation of *Lavandula dentata* resulted in 3.47% of characteristic EO. This yield is higher in comparison with that obtained from Tunisian lavender (0.89%) [22], and also exceeded that of the same plant collected from other regions of Morocco (2.9%) [23]. The impact of various environmental conditions can be responsible for the variation in extraction yield across plants [24]. Linalool (49.71%), followed by camphor (14.36%) and borneol (8.21%), were the major components identified in *Lavandula dentata* EO by GC-MS analysis. Other chemotypes of lavender in Morocco were reported to be rich in 1.8 cineol (41.28%) in eastern Morocco [25], while those collected in the Moroccan Middle Atlas were marked by high camphor content (49.75%) [26]. The EO extracted from the leaves of Tunisian *L*. *dentata* was characterized by the dominance of camphor (26.52%) and 1.8 cineole (22.90%) [22], followed by *β*-eudesmol (21.17%), myrtenol (13.02%), and sabinol (11.02%) [27]. Similarly, the 1,8 cineole (63.25%) was also found to be the major component of the EO of lavender growing in Brazil [28]. Carvacrol (14.82%), terpinen-4-ol (5.98%), *α*-pinene (2.57%), and linalool (0.54%) compounds were also found in *N. sativa* (black Caraway) EOs [29]. Camphor, *α*-pinene, and terpinen-4-ol were also reported among the compounds figured in the EO of Tunisian and French fennel (*F. vulgare* Mill.) [30]. In addition to the method of extraction, the difference in the composition of EOs can be related to different factors, including circadian rhythms, seasonal conditions, and environmental impacts [28]. Many compounds in the characterized EO such as linalool, borneol, camphor, carvacrol, and thymol were reported to be potentially implicated in several biological activities [31].

Then, *in vitro*, the antioxidant capacities of *Lavandula dentata* EO and linalool compound to limit lipid oxidation were assessed, in this study, using the *β*-carotene assay. As multiple studies showed that fatty acid oxidation represents one of the principal causes of food deterioration. Natural food preservatives are frequently used to reduce lipid oxidation [32]. The obtained results demonstrated that lavender EO possessed the ability to limit lipid oxidation. This inhibitory ability increased with increasing concentrations (Figures 2 and 3). Several studies reported a moderate antioxidant effect of *Lavandula dentata* EO using various tests [28, 33, 34]. The antioxidant potential of Tunisian *Lavandula dentata* was also proved, by recording IC_50_ values of 53.029 mg/mL, 113.29 mg/mL, and 43.20 mg/mL, respectively, for the ABTS test, the DPPH test, and the reducing power test [27]. Our results are more promising than those of other spices of the lavender plant such as *L. officinalis, L. stoechas,* and *L*. *pedunculata* which displayed lesser lipid peroxidation inhibition activity that did not exceed 52.52 *±* 2.01%, 14.8 *±* 3.1%, and 17.2 *±* 1.3%, respectively [35, 36]. The capacity of lavender EO to inhibit lipid peroxidation is probably due to the combination of molecules it contains and the high proportion of unsaturated compounds and oxygenated compounds (91.07%), which have distinct polarity and functional groups, resulting in diverse chemical behavior [32]. On the other hand, linalool as a major component of the lavender EO, showed better antioxidant capacity, which was very close to that of BHT at high concentrations. These findings suggest that this compound may be the most implicated in antioxidant activity. According to some research studies on the antioxidant capacity of many chemicals found in our investigated plant, linalool has a higher antioxidant potential than camphor, 1.8 cineole, borneol, and terpinene-4-ol in several tests. Whereas, thymol and carvacrol displayed a higher antioxidant activity [31]. Consequently, this wide variety of molecules in the examined EO, as well as their probable action modes and interactions, makes it difficult to assign the antioxidant activity to just one or only a few active components. Nowadays, common antioxidant tests, such as inhibition of linoleic acid oxidation, could provide important information about the activities of molecules of interest and how food products oxidize. In modern biotechnology, EOs and isolated phenolic compounds as potential additives (as effective antioxidants with low toxicity) have been employed in various forms, both free and encapsulated, in nondegradable and biodegradable materials [8]. Moreover, and through several tests, numerous studies have evaluated the antioxidant capacity using different food matrices by combining various films and EOs. The results were promising [8]. Therefore, the remarkable antioxidant potential of EOs extracted from aromatic plants such as *L. dentata* makes them promising alternatives to synthetic food preservatives.

Then, *in silico*, molecular docking of the antioxidant activity of *Lavandula dentata* EO against NADPH oxidase was also examined in this work. Thymol, carvacrol, *α*-terpineol, and camphor were the most active lavender compounds exhibiting an inhibitory effect against NADPH oxidase. In fact, NADPH oxidase is a major enzymatic source of oxygen-free radicals in stimulated endothelial cells [37]. The inhibition of this protein represents a major key in the protection of cells against free radicals. Several *in vitro* studies have reported the antioxidant activity of lavender EO [7, 27]. Our results are in agreement with a study where derivatives of thymol and carvacrol exhibited remarkable antioxidant activity according to 1,1-diphenyl-2-picrylhydrazyl assay and Rancimat assay [38]. Another study noted high antioxidant activity *in vitro* of camphor extract using the DPPH test [39]. The obtained results demonstrate that EOs from *Lavandula dentata* may constitute natural NADPH inhibitors.

The antibacterial activities of *Lavandula dentata* EO and linalool were evaluated, qualitatively and quantitatively, against food-borne pathogenic bacteria. Results showed that lavender EO was generally active against a broad spectrum of food intoxication isolated strains including both Gram (−) bacteria (*S. enterica, E. coli,* and *P. aeruginosa*) and Gram (+) (*S. aureus* and *B. subtilis*) bacteria. Our data confirm previous conclusions and attest, in some cases, to the superiority of our results [25, 28, 34]. These investigations reported the antibacterial activity of *Lavandula dentata* against a wide spectrum of bacteria including *E. coli, S. aureus, S. pneumoniae, L. monocytogenes, K. pneumoniae, P. aeruginosa, S. pyogenes, N. meningitidis,* and *Salmonella* sp. Similarly, EOs extracted from *Lavandula dentata* of southern Morocco were likewise found to be effective against *E. coli* and *S. aureus* by marking characteristic inhibition zones of 12 ± 0.29 mm and 30 ± 0.00 mm and MIC values of 0.14 mg/mL and 0.08 mg/mL, respectively [23]. Tunisian *Lavandula dentata* essences were also significantly active against six bacterial strains, including *P. aeruginosa*, *S. aureus,* and *E. coli*, where MIC values ranged between 0.1 and 0.6 *μ*g/mL [27]. The antibacterial activity of lavender EO was confirmed in another study against *E. coli* and *S. aureus* with low MIC values ranging between 2000 ppm and 1000–1200 ppm, respectively [40]. Likewise, recent works showed that the application of *Lavandula stoechas* EO at a concentration of 60 *μ*L of EO/disc totally inhibited the growth of *Staphylococcus aureus* strains and exhibited a marked action on the growth of the majority of Gram (+) bacteria tested [41]. The probable mechanisms of the antimicrobial power of lavender EOs especially against Gram (+) bacteria may be related to the direct interaction of their hydrophobic components with the cell membrane, and their power to partition bacterial phospholipids by making them more permeable, which may result in structural damage or total rupture of cell membranes and loss of nutrients [23]. Gram (−) bacteria possess an outside lipopolysaccharides membrane, which can limit the diffusion of hydrophobic components [18]. Moreover, research studies on terpenes' effects on the isolated bacterial membrane suggested that the high potential of EOs is due to the lipophilic properties of terpenes, the strength of their functional groups, and their aqueous solubility. Several processes including protein translocation, phosphorylation, inhibition of electron transport, and other enzymatic reactions may also be involved [23]. Several reports have shown the significant antimicrobial potential of some EO's major compounds such as thymol, carvacrol, camphor, linalyl acetate, 1.8-cineole, and terpinen-4-ol against many foodborne pathogen bacterial strains (*E. faecalis*, *P. aeruginosa*, *B. cereus*, *E. coli*, and *S. aureus*) and against lactic acid bacteria, especially against *L. acidophilus* [31, 42]. Linalool was the major compound of our investigated EO. This unsaturated monoterpene with a phenolic ring demonstrated in the current study that it possessed antimicrobial potential that is comparable to and close to that of the EO against five food-borne pathogenic bacterial strains, attesting to its significant contribution to the antibacterial activity of lavender EO. In general, phytochemicals in the EO may act synergistically rather than individually, as numerous studies showed that the antimicrobial effect of EOs exceeded sometimes that of its single compounds tested separately [31, 43]. Since 2000, the number of antibiotics recommended for clinical usage has decreased and the majority of them are only effective against gram (+) bacteria, while their effectiveness against Gram (−) strains decreases with time, which may constitute a real threat to human health [32]. Therefore, the restricted treatment strategies for drug-resistant pathogens require the development of new and more effective alternatives. Moreover, in the field of food preservation and packaging, the current tendency is to use natural substances instead of chemically synthesized ones. Lavender EO has already been tested as a food preservative. In this context, a previous study showed that the incorporation of chitosan beads containing lavender EO at 2 concentrations (0.125 and 0.25 g/sachet) into strawberry packed in clamshell can effectively inhibit *B. cinerea* growth. Experiments showed also a maintenance of appearance, color, and firmness [44]. Consequently, the authors recommended the application of lavender EOs inside the packaging system to promote an antifungal activity against grey mold rot on strawberry fruit [45, 46]. Therefore, *L. dentata* EO can be a promising agent to combat food-borne pathogens and it can also constitute as a good food preservative.

## 5. Conclusions

In conclusion, the findings of the present study confirm that the essential oils extracted from Moroccan *L*. *dentata*, through their bioactive chemical compounds such as linalool, exhibit interesting antimicrobial activity against a spectrum of food-borne pathogens, in addition to their high capacity to limit lipid peroxidation, and to inhibit NADPH oxidase enzymatic complex in stimulated endothelial cells. These results suggest promising usage of these essential oils in the treatment of food-borne microbial infections, as well as in the food industry and packaging systems as alternatives to synthetic food preservatives. However, further research studies dealing with toxicities on humans and nontarget organisms is needed before any application.

## Figures and Tables

**Table 1:** Chemicals identified in *L. dentata* EO.

RT (min)	RI	Compounds	Formula	Content (%)	Chemical structure
7.922	948	*α*-Pinene	C_10_H_16_	0.91	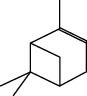

10.828	1059	1,8-Cineole	C_10_H_18_O	5.47	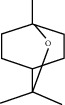

11.971	1164	cis-Linalool oxide	C_10_H_18_O	0.98	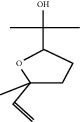

12.450	1164	trans-Linalool oxide	C_10_H_18_O	0.67	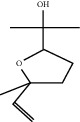

12.824	1082	Linalool	C_10_H_18_O	49.71	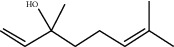

14.278	1121	Camphor	C_10_H_16_O	14.36	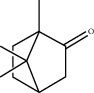

14.735	1146	Lavandulol	C_10_H_18_O	1.13	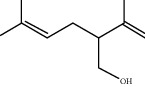

15.019	1138	Borneol	C_10_H_18_O	8.21	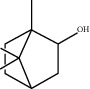

15.269	1137	Terpinen-4-ol	C_10_H_18_O	7.05	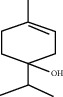

15.421	1112	cis-Ocimenone	C_10_H_14_O	0.42	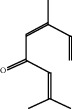

15.530	1183	*n*-Hexyl butanoate	C_10_H_20_O_2_	0.52	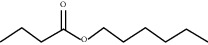

15.678	1143	*α*-Terpineol	C_10_H_18_O	1.11	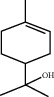

17.197	1272	Linalyl acetate	C_12_H_20_O_2_	4.81	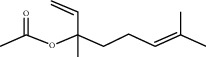

18.133	1270	Lavandulyl acetate	C_12_H_20_O_2_	1.00	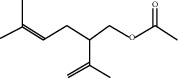

18.319	1262	Thymol	C_10_H_14_O	1.71	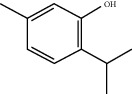

18.557	1262	Carvacrol	C_10_H_14_O	0.78	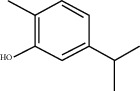

22.642	1440	*β*-Farnesene	C_15_H_24_	0.67	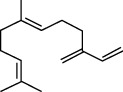

23.846	1504	(R)-Lavandulyl (R)-2-methylbutanoate	C_15_H_26_O_2_	0.46	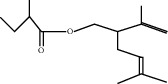

*Chemical classes (%)*					
Monoterpene hydrocarbons				92.51	
Sesquiterpene hydrocarbons				0.67	
Other compounds				6.79	
Total (%)				99.97	

**Table 2:** Docking results of *L. dentata* EO in the active site of NADPH (PDB: 2CDU).

EO compounds	Glide score (kcal/mol)	Glide Emodel (kcal/mol)	Glide energy (kcal/mol)
Thymol	−6.483	−33.124	−23.764
Carvacrol	−6.17	−32.53	−23.46
*α*-Terpineol	−4.728	−25.282	−19.396
Camphor	−4.541	−28.001	−21.354
Terpinen-4-ol	−4.483	−23.15	−18.4
Borneol	−4.362	−27.569	−21.336
Lavandulyl acetate	−3.841	−33.464	−30.354
*α*-pinene	−3.763	−18.56	−15.108
cis-Linalool oxide	−3.689	−29.897	−25.14
trans-Linalool oxide	−3.634	−35.376	−29.279
Cineole	−3.56	−23.689	−19.321
Linalool	−2.951	−23.6	−20.027
cis-Ocimenone	−2.87	−22.441	−19.37
Linalyl acetate	−2.653	−24.182	−21.123
Lavandulol	−2.25	−21.265	−19.572
(R)-Lavandulyl (R)-2-methylbutanoate	−0.919	−24.344	−25.495
*n*-Hexyl butanoate	0.395	−22.499	−24.571
*β*-Farnesene	0.422	−20.402	−21.975

**Table 3:** Inhibition zone diameter (mm) of linalool and EO extracted from *L. dentata* against bacterial strains (means ± SEM).

	*Inhibition diameter (mm)*				
	*Gram-negative bacteria*			*Gram-positive bacteria*	
	*E. coli* (CIP 53126)	*P. aeruginosa* (CIP 82118)	*S. enterica* (CIP 8039)	*B. subtilis* (CIP 5262)	*S. aureus* (CIP 483)
Lavender EO	17.08 ± 0.79^b^	9.06 ± 0.63^c^	15.16 ± 0.72^c^	15.08 ± 0.65^c^	15.08 ± 1.22^c^
Linalool	20.08 ± 0.65^b^	15.08 ± 0.50^b^	23.08 ± 1.22^b^	29.00 ± 1.15^b^	25.00 ± 1.73^b^
Streptomycin	NI	9.00 ± 0.57^c^	—	10.08 ± 1.22^c^	NI
Vancomycin	NI	12.91 ± 0.50^bc^	—	—	14.08 ± 0.65^c^
Chloramphenicol	35.08 ± 1.80^a^	34.00 ± 1.44^a^	36.91 ± 1.80^a^	35.00 ± 2.30^a^	36.83 ± 1.87^a^

NI : no inhibition. In every column, values with different letters are significantly different (*p* < 0.05).

## Data Availability

The data used to support the findings of the study are included within the article.
